# Bio-Gel Formation Through Enzyme-Induced Carbonate Precipitation for Dust Control in Yellow River Silt

**DOI:** 10.3390/gels11060452

**Published:** 2025-06-12

**Authors:** Jingwei Zhang, Hualing Jia, Jia Li, Xuanyu Chen, Lei Wang, Shilong Wang, Lin Liu

**Affiliations:** 1 School of Civil Engineering, Zhengzhou University, Zhengzhou 450001, China; zhangjingwei@zzu.edu.cn (J.Z.); jhl969517830@163.com (H.J.); lwang029@zzu.edu.cn (L.W.); 2 School of Water Conservancy and Transportation, Zhengzhou University, Zhengzhou 450001, China; 3 School of Transportation, Southeast University, Nanjing 211189, China; cxyyes@163.com; 4 Zhengzhou City Construction Group Investment Co., Ltd., Zhengzhou 450007, China; 24384002@163.com (S.W.); 524175404@163.com (L.L.)

**Keywords:** EICP, dust control, wind and rain resistance, wear resistance, bio-cemented crust

## Abstract

This study explored the enzymatic formation of gel-like polymeric matrices through carbonate precipitation for dust suppression in Yellow River silt. The hydrogel-modified EICP method effectively enhanced the compressive strength and resistance to wind–rain erosion by forming a reinforced bio-cemented crust. The optimal cementation solution, consisting of urea and CaCl_2_ at equimolar concentrations of 1.25 mol/L, was applied to improve CaCO_3_ precipitation uniformity. A spraying volume of 4 L/m^2^ (first urea-CaCl_2_ solution, followed by urease solution) yielded a 14.9 mm thick hybrid gel-CaCO_3_ crust with compressive strength exceeding 752 kPa. SEM analysis confirmed the synergistic interaction between CaCO_3_ crystals and the gel matrix, where the hydrogel network acted as a nucleation template, enhancing crystal bridging and pore-filling efficiency. XRD analysis further supported the formation of a stable gel-CaCO_3_ composite structure, which exhibited superior resistance to wind–rain erosion and mechanical wear. These findings suggest that gel-enhanced EICP represents a novel bio-gel composite technology for sustainable dust mitigation in silt soils.

## 1. Introduction

**Figure 1:**
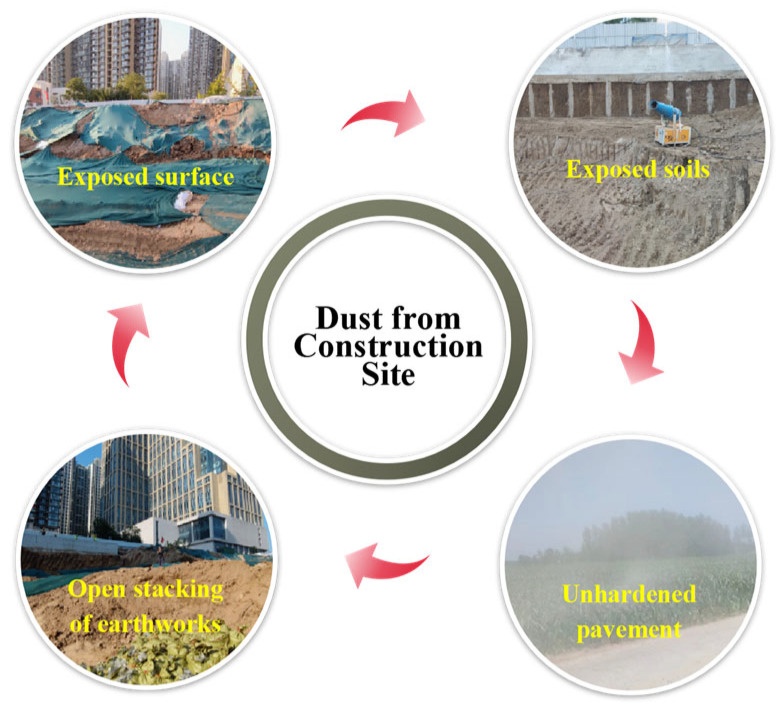
Dust source from construction site.

The soil composition in the alluvial plains of the Yellow River in China mainly consists of silt, characterized by low water stability and challenging compaction, leading to the easy generation of dust during construction activities, thereby posing a substantial threat to air quality [1]. The main source of dust generation is construction sites (refer to Figure 1), including exposed surfaces, open earth stacking, and delayed road surface hardening. Conventional dust suppression techniques such as water sprinkling, dust inhibitors, and dustproof nets exhibit specific drawbacks, including their temporary effectiveness [2,3], stringent application requirements [4], and the potential for inducing secondary pollution.

For the past several years, to address the shortcomings of traditional dust suppression methods, microbially induced carbonate precipitation (MICP) technology has been extensively studied and applied in engineering to enhance the wind erosion resistance of soil [5,6,7]. However, due to limitations such as the size, aerobic nature, and cost of urease-producing bacteria, the MICP technology has restrictions on the applicable soil types and soil depth [8,9]. Consequently, researchers have proposed enzyme-induced carbonate precipitation (EICP) technology (as shown in Formula (1) and (2)), which offers the following advantages compared to MICP technology: the small size of free urease can penetrate fine-grained soils [10,11], the reaction can be completed without the need for nutrients or air supply [12,13], and the degradability of urease avoids long-term environmental impacts [14]. Researchers have extracted urease from various plant seeds, such as beans [15,16], watermelon seeds [17], and soybeans [18,19], to address the issue of expensive commercial urease prices [20]. Among them, soybeans have become ideal research subjects due to their widespread cultivation in Asia and extensive application in the food industry. Previous studies have confirmed the feasibility of using soybean urease in EICP technology [18,21,22]. The effectiveness of EICP technology in soil solidification is influenced by various factors, including urease activity [23,24], grouting solution concentration [25], environmental conditions [26,27,28], and construction processes [10,29]. Despite numerous experimental studies on EICP technology, the formulation of EICP dust suppressants for silt soils in the Yellow River alluvial plain region remains undetermined, and the impact of EICP construction processes on the solidification effects of silt soils remains unclear. Understanding of the wind, rain, and wear resistance of the anti-dust solidification layer is still lacking.

(1) $$CO(NH_{2}{)}_{2}+2H_{2}O\overset{urease}{\to }CO_{3}^{2-}+2NH_{4}^{+}$$

(2) $$Ca^{2+}+CO_{3}^{2-}\to CaCO_{3}↓$$

The objective of this study is to investigate the effectiveness of soybean urease-induced carbonate precipitation in suppressing dust in a typical silt soil environment within the Yellow River alluvial plain region. Through a gradient test and optimization of the EICP dust suppressant formulation, various factors affecting urease activity and curing efficiency were elucidated, along with an investigation into the underlying mechanism for dust suppression. Based on these findings, a construction process for EICP dust suppression was determined, and characteristics such as wear resistance, wind resistance, and rain resistance were evaluated.

## 2. Results and Discussion

### 2.1. Optimization of EICP Solution

#### 2.1.1. Particle Size and Solid–Liquid Mass Ratio

**Figure 2:**
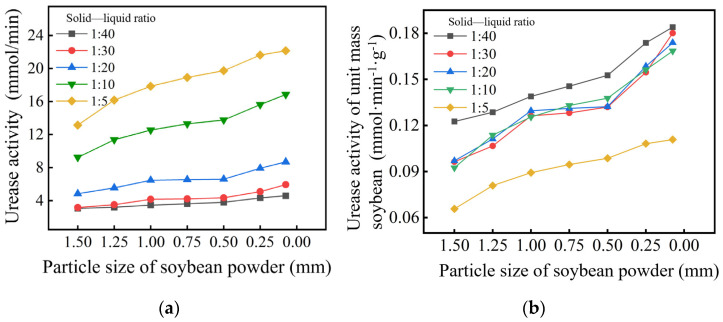
Effect of the nature of soybean powder on urease activity: (**a**) the urease activity; (**b**) the urease activity of unit mass soybean.

According to Figure 2a, when the particle size is 0.50 mm, the urease activity at a solid–liquid ratio of 1:5 is 143.30% higher than that at a ratio of 1:10, and 517.19% higher than that at a ratio of 1:40, indicating a significant increase in urease activity with an increasing solid–liquid ratio. Unit mass urease activity reflects the extraction efficiency of urease. As shown in Figure 2b, the urease extraction efficiency is highest at a solid–liquid ratio of 1:40, remains relatively consistent within the range of 1:30 to 1:10, and significantly decreases at a ratio of 1:5. At a particle size of 0.50 mm, the unit mass urease activity at a solid–liquid ratio of 1:5 is 64.47% of that at a ratio of 1:40 and 71.53% of that at a ratio of 1:10. This urease activity curve can approximately characterize the yield of calcium carbonate precipitation. Higher urease activity accelerates carbonate availability, thereby enhancing precipitation kinetics [9,10].

In summary, the observed increase in urease activity with smaller soybean powder particle sizes (<0.50 mm) is attributed to the larger specific surface area of finer particles. Reduced particle size enhances the exposure of encapsulated urease enzymes, facilitating more efficient substrate (urea) diffusion and active site accessibility. When the solid–liquid ratio is low (1:40), the total urease content in the soybean urease solution may be insufficient, resulting in lower urease activity and failing to meet experimental requirements. Conversely, when the solid–liquid ratio is high (1:5), the soybean powder solution has a high oil content and viscosity, making infiltration into fine-grained soil difficult and resulting in low urease extraction efficiency. Therefore, considering the requirements for urease activity and cost-effectiveness in engineering applications, this study recommends preparing urease solutions with particle sizes less than 0.50 mm and a solid–liquid ratio of 1:10.

#### 2.1.2. pH Value

**Figure 3:**
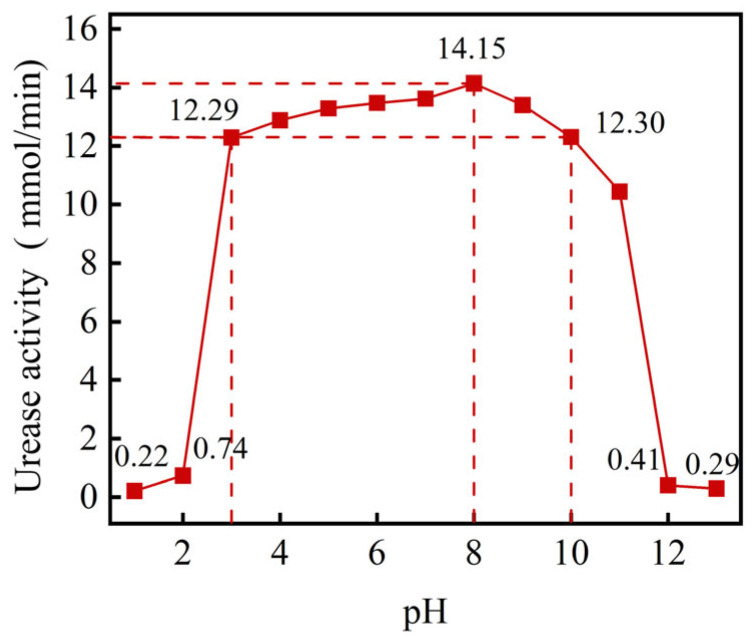
The urease activity at different initial pHs.

The activity of soybean urease demonstrates an initial increase followed by a decrease as the initial pH rises, as depicted in Figure 3. Notably, the urease activity reaches a notable level of 13.40~14.15 mmol/min when the solution’s pH ranges between 7 and 9 and reaches peaks at pH 8. Conversely, urease activity diminishes significantly when the solution’s pH falls below 2 or exceeds 12. This decline can be attributed to the urease’s proteinaceous nature, which renders it susceptible to structural damage under extremely acidic or alkaline conditions, consequently leading to its inactivation. The PH value mainly affects the activity of urease. When the PH is 8, the urease has a relatively high activity, and it can be approximately considered that the yield of calcium carbonate is better at this time. Hence, the optimal activity of the urease solution is discerned at an initial pH of 8.

#### 2.1.3. Concentration and Volume Ratio of Reaction Solution

**Figure 4:**
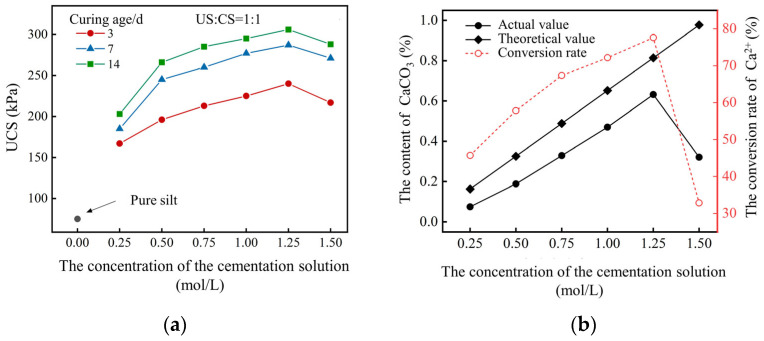
Influence of concentration of CS on solidification effect: (**a**) UCS; (**b**) the content of CaCO_3_.

Unconfined compressive strength tests were conducted on silty soil samples treated with different concentrations of cementation solutions. Portions of soil samples from the fractured surfaces were taken to measure the calcium carbonate content; the experimental results are shown in Figure 4.

From Figure 4a, it can be observed that the unconfined compressive strength of the soil sample increases with the curing age. When the concentration of the cementation solution was 1.25 mol/L, the unconfined compressive strength of the sample cured for 3 days was 240 kPa, which was 83% of the strength at 7 days and 78% of the strength at 14 days. A gradual increase in the unconfined compressive strength of the soil sample was observed when the concentration of the cementation solution increased from 0.25 mol/L to 1.25 mol/L. However, the unconfined compressive strength of the soil sample decreases when the concentration of the cementation solution exceeds 1.25 mol/L. The UCS value approximately indirectly reflects the precipitation content of calcium carbonate. The higher the value, the higher the precipitation yield of calcium carbonate. From Figure 4b, it can be seen that as the concentration of the cementation solution increases from 0.25 mol/L to 1.25 mol/L, the content of CaCO_3_ in the soil sample gradually increases, reaching a maximum at a cementation solution concentration of 1.25 mol/L, accounting for 0.63% of the total mass of the soil and the conversion rate of Ca^2+^ reaching 77.61%. The presence of excessive plant proteins in the crude soybean urease solution can impede the precipitation of CaCO_3_. Therefore, a moderate increase in the concentration of the cementation solution will increase the content of CaCO_3_. However, as the concentration of the cementation solution further rises, the elevated levels of calcium ions therein suppress the activity of soybean urease and diminish the efficacy of urea hydrolysis [30]. This conclusion is consistent with the research results of Carmona et al. [31].

**Figure 5:**
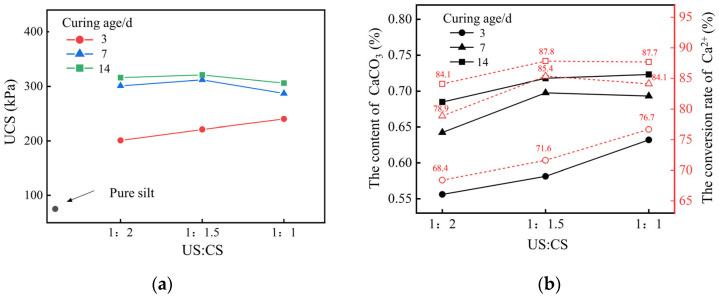
Influence of volume ratio on solidification effect: (**a**) UCS; (**b**) the content of CaCO_3_.

The Impact of the volume ratio of urease to cementation solution on the EICP effect was examined by measuring the unconfined compressive strength (UCS) of soil samples at various curing stages. Subsequently, soil samples taken from the fractured surfaces after UCS testing were analyzed for calcium carbonate content. The experimental findings are presented in Figure 5.

As shown in Figure 5, at a 3-day curing age, there was a linear increment in UCS as the volume ratio of urease solution to cementation solution increased. Conversely, over the 7- and 14-day curing ages, UCS initially rose with the volume ratio and then decreased. Based on the literature [32], it is evident that as biochemical reactions progress, urease in the soil will gradually lose its activity, reducing the production of CaCO_3_. Therefore, considering solidification effectiveness and economic factors, the optimal concentration of the cementation solution should be set at 1.25 mol/L, and the volume ratio of urease to cementation solution was 1:1.

### 2.2. Construction Technology

Spraying solution is the most cost-effective and practical method for mitigating dust issues on sites with silty soil. This approach leverages the solution’s penetrating ability in the soil to promote calcium carbonate precipitation among the silty soil particles, leading to the formation of a solidification layer aimed at achieving effective dust control.

#### 2.2.1. Influence of Spraying Scheme on Solidification Effect

**Figure 6:**
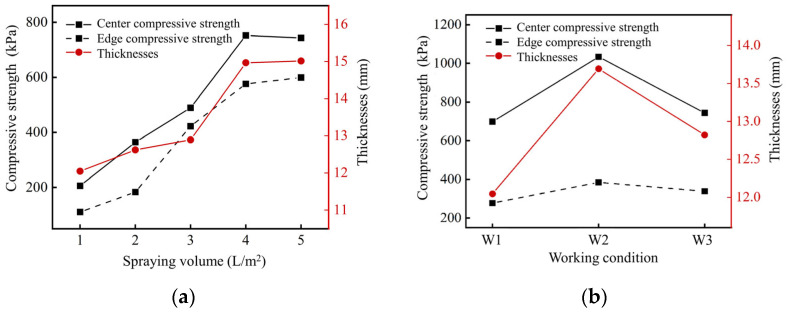
Influence of spraying scheme on solidification effect: (**a**) different spraying volumes on solidification effect; (**b**) different spraying methods on solidification effect.

Figure 6a illustrates the solidification performance of silt specimens subjected to varying spraying volumes and spraying methods. Initially, with a small spraying volume, the EICP solution failed to penetrate the soil thoroughly, thereby resulting in the formation of a thin solidification layer on the surface and limited curing effect of the silt. As the spraying volume increased, the solution penetrated the silt more effectively, promoting the formation of bio-gel materials through EICP that subsequently enhanced the compressive strength of the specimens. Notably, at a spraying volume of 4 L/m^2^, the surface compressive strength of the samples increased to 752.20 kPa, with a solidification layer thickness of 14.97 mm, demonstrating the effective gelation capacity of EICP-generated cementitious materials. Nevertheless, further increases in spraying volume led to marginal improvements in surface compressive strength and solidification-layer thickness. This phenomenon could be attributed to the formation of calcium carbonate precipitation and subsequent bio-gel network development among soil particles on the surface as the EICP solution volume increased, hindering additional penetration and reaching a critical solidification value. As a result, a spraying volume of 4 L/m^2^ was deemed optimal for subsequent experimental spraying procedures.

Working conditions W1, W2, and W3 are shown in Table 1. From Figure 6b, it can be seen that the solidification effects of W1 and W3 were both unsatisfactory. In these conditions, both urease and urea solution were pre-mixed, which means that the hydrolysis reaction of urea had already begun before the EICP solution was sprayed onto the soil surface. This premature formation of CaCO_3_ created a dense bio-gel layer that binds with soil particles, hindering the penetration of the solution into deeper layers of the silt. In contrast, the EICP solution demonstrates optimal permeation and diffusion into the deep silt in condition W2. After the urease solution was injected, the controlled gelation process allowed the hydrolysis and precipitation reactions to proceed gradually, resulting in a uniform bio-cemented gel matrix with a thicker solidification layer (14.97 mm) and higher soil compressive strength (752.20 kPa) compared to other working conditions. This sequential spraying strategy optimized the in situ gel formation, demonstrating the effectiveness of EICP-generated cementitious materials. Therefore, in subsequent experiments, the practice of initially spraying a mixture of calcium chloride and urea solutions followed by urease solution was maintained.

#### 2.2.2. Reaction Time

**Figure 7:**
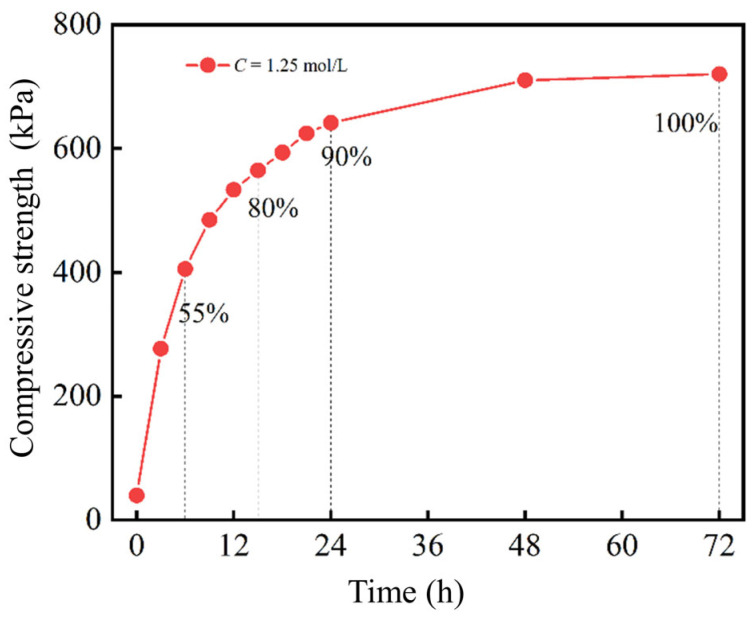
Influence of reaction time on solidification effect.

Figure 7 shows the relationship between time and solidification. The solidification effect increases rapidly in the initial stage of the reaction due to the rapid formation of bio-gel structures through EICP-induced CaCO_3_ precipitation. The solidification effect can reach 80% at the 15 h mark and further increase to 90% within 24 h, with the surface strength of specimens cured for 72 h serving as the reference benchmark. As the reaction progresses, a bio-cemented gel layer composed of calcium carbonate and silt develops on the specimen surface, whose dense gel-like microstructure halts the further penetration of the EICP solution into the deep silt. The incompletely reacted solution in the deep layers of the silt further precipitated to form CaCO_3_ until the reaction reached completion, demonstrating the self-limiting nature of EICP gelation. Consequently, the reaction exhibits rapid progress in the initial stage, with a gradual decrease in the reaction rate over time as the bio-gel network matures and stabilizes. In practical engineering applications, the reaction time can be adjusted according to the desired curing effect and time requirements to optimize the balance between gelation efficiency and material penetration depth, thereby achieving optimal benefits.

### 2.3. Curing Effect

#### 2.3.1. Macro Appearance

**Figure 8:**
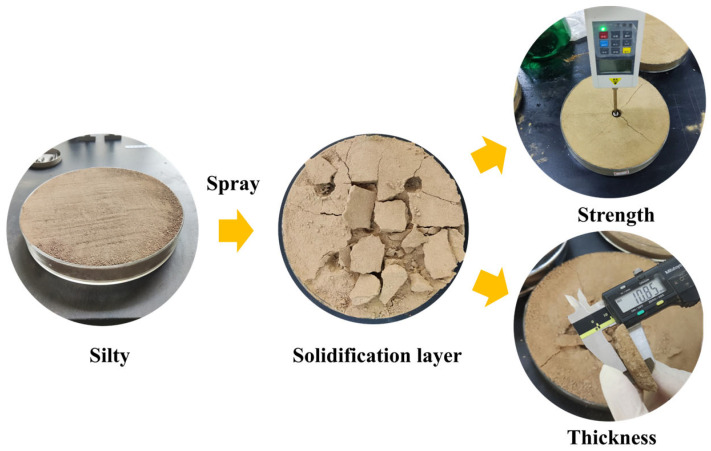
Soil crust induced by EICP.

Spraying EICP solution onto the surface of silt soil can increase the calcium carbonate content and induce the formation of a soil crust through bio-gel bonding, thereby enhancing the cohesion among soil particles and improving surface strength. As depicted in Figure 8, a calcareous layer, namely a bio-cemented gel layer with a dense microstructure, developed, and its thickness serves as one of the evaluation indicators for assessing curing effects. Using a vernier caliper, as illustrated in Figure 8, measurements were taken to determine the thickness of the soil crust, with its maximum value reaching 15.01 mm, demonstrating the structural stability of the EICP-derived gel matrix.

#### 2.3.2. Micro Analysis

**Figure 9:**
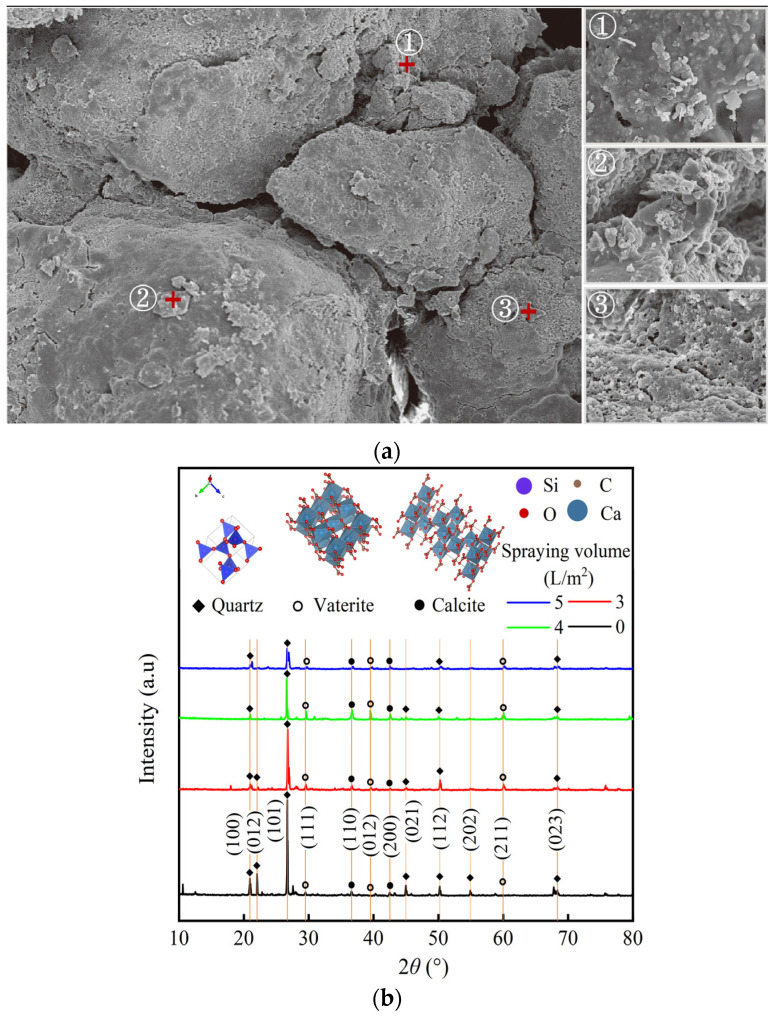
Micro analysis results: (**a**) SEM image of specimen after EICP treatment; (**b**) XRD pattern of silt specimens.

The SEM and XRD results of the soil sample after EICP treatment are shown in Figure 9.

1. SEM

Figure 9a reveals spherical or globular aggregates of irregular size induced by soybean urease. The interaction between soil particles and crystals can be categorized into three types: ① film covering, where crystals cover the surface of soil particles, increasing particle volume and surface area without contact with other soil particles; ② cementation, where crystals primarily precipitate and aggregate near contact points of particles, acting as a bonding agent; ③ bridging, involving the precipitation and aggregation of crystals between neighboring particles to ultimately link them together. These three modes of crystal cementation effectively reduce soil porosity and permeability, while the structural durability and strength rely on crystals that perform cementation and bridging functions. The results confirm the positive effect of EICP on silt solidification.

2. XRD

The distribution of phase composition in silt specimens treated with EICP is depicted in Figure 9b. As the spraying volume increased, there were discernible shifts in the diffraction peak values of crystals, indicative of changes in the relative content of crystal components. Notably, the peak intensities associated with the crystal faces of quartz (SiO_2_), such as (100), (012), (101), (021), (112), (202), and (023), have all decreased. Particularly, at a diffraction angle of 26.7°, the peak intensity of the crystal face (101) decreased from 3985 at a spraying volume of 0 L/m^2^ to 1434, 339, and 717 at spraying volumes of 3, 4, and 5 L/m^2^, respectively. Moreover, in crystal faces representing vaterite (111), (012), (211), and calcite (110), (200), the corresponding diffraction peak intensities increased to varying degrees with the increase in spraying volume. Compared to the untreated silty soil samples, it is evident that the silty soil samples treated with EICP technology exhibit distinct diffraction peaks of calcite and chondraronite. Calcite, being the most thermodynamically stable mineral phase in calcium carbonate polycrystalline form, possesses good mechanical strength capable of cementing silt particles, thereby enhancing the structural stability of silt. Consequently, the compressive strength of silt samples significantly improves after EICP treatment.

### 2.4. Dust Control Effect

#### 2.4.1. Wind–Rain Resistance

**Figure 10:**
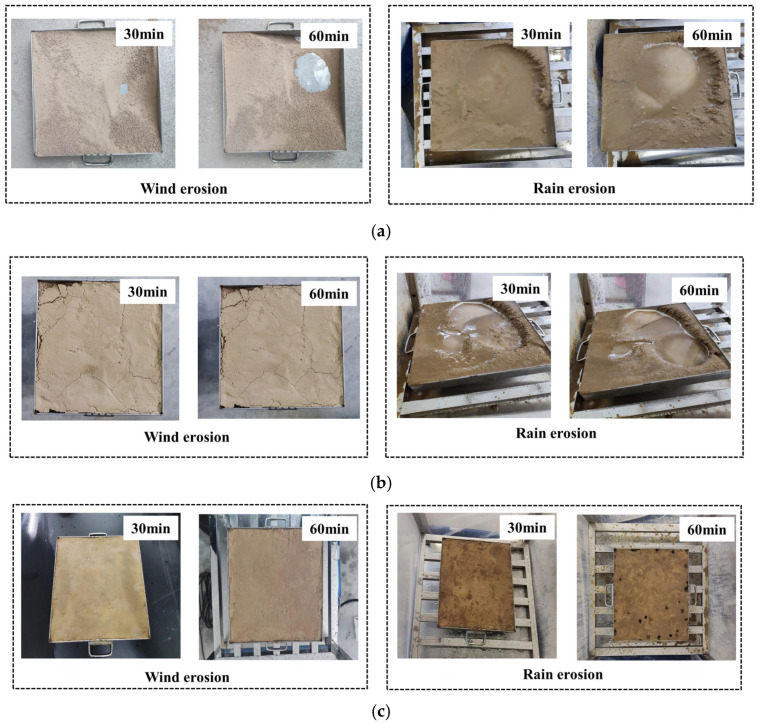
Morphology analysis of wind and rain erosion: (**a**) untreated specimens; (**b**) deionized water treated specimens; (**c**) EICP treated specimens. In contrast, the untreated specimen experienced a significantly higher mass loss of 2459.38 g under similar conditions. For rainfall erosion, after 60 min at a rate of 40 mm/h, the EICP-treated soil specimen showed a rain erosion coefficient of 1.39%, with a resulting mass loss of 83.50 g. In comparison, the untreated soil specimen lost 1095.63 g in mass under the same rainfall conditions.

As shown in Figure 10, significant morphological changes occurred in the untreated soil sample within 30 min under wind erosion, resulting in substantial soil particle loss and extensive exposure of the mold bottom. The soil sample treated with deionized water exhibited continued soil particle loss, primarily evidenced by the widening of surface cracks. Furthermore, this sample experienced substantial soil particle loss during rain erosion, leading to notable alterations in surface morphology. The specimens treated with EICP showed minimal morphological alterations following wind erosion due to the bio-gel-reinforced crust formed through CaCO_3_ crystal networking. After 60 min of rain erosion, the gel-like cementation matrix resulted in relatively minor morphological changes. Comparing the morphological alterations among the specimens indicates that the EICP-generated bio-cement gel exerts a discernible inhibitory effect on dust emission in silt.

**Figure 11:**
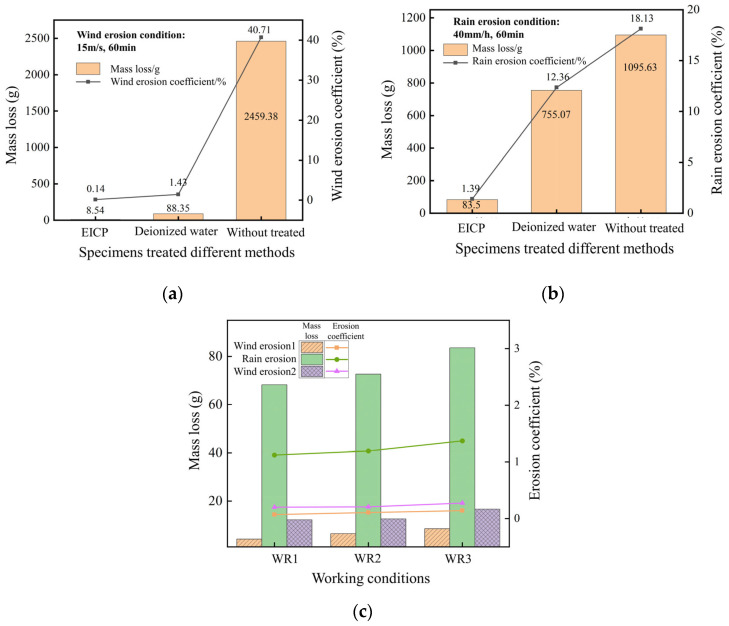
Test results of wind and rain erosion: (**a**) mass loss of wind erosion test; (**b**) mass loss of rain erosion test; (**c**) mass loss of different working conditions.

Figure 11a,b illustrate the results of wind and rain erosion tests on EICP-treated and untreated soil specimens. After 60 min of wind erosion at a wind speed of 15 m/s, the EICP-treated specimen exhibited a wind erosion coefficient of 0.14%, resulting in a mass loss of 8.54 g.

Figure 11c shows the mass loss and erosion coefficients of silt specimens treated with EICP under different working conditions, as listed in Table 1. In the first round of wind erosion tests with wind speeds of 60 min, the bio-gel-reinforced specimens exhibited mass loss less than 8.54 g (accounting for <0.2% of total mass), demonstrating the cohesive strength of the CaCO_3_-gel composite matrix. After rainfall erosion tests for 60 min, the gel-stabilized samples showed mass loss below 83.50 g (~1.2% of total mass), attributed to the water-resistant nature of the bio-mineral gel network. Following drying, the persistent gel-phase cementation maintained dust suppression ability, with second-round wind erosion mass loss not exceeding 16.66 g per group. These results indicate that the EICP-derived gel-cemented layer can resist Beaufort scale 7 winds (51–61 km/h) and 40 mm/h rainfall rates, where the self-healing gel architecture prevents crack propagation under cyclic wet–dry conditions.

These findings align closely with Sun et al. [11], who demonstrated that EICP-PVAc (50 g/L) treatment achieved surface strengths exceeding 500 kPa and near-zero soil loss under extreme rainfall (300 mm/h). Field tests by Sun et al. revealed CaCO_3_ crystallization within 10–27 mm thick cemented layers, corroborating the cohesive mechanism documented in the present study. While Sun et al. focused on slope-scale stabilization under high-intensity rainfall, the current investigation extends these insights by quantifying the resistance of EICP-gel composites to cyclic wind and rainfall stresses. This advancement builds upon previous methodologies through the systematic evaluation of long-term durability under combined environmental stressors, addressing a critical gap in prior EICP applications focused solely on static hydrological conditions.

#### 2.4.2. Wear Resistance

**Figure 12:**
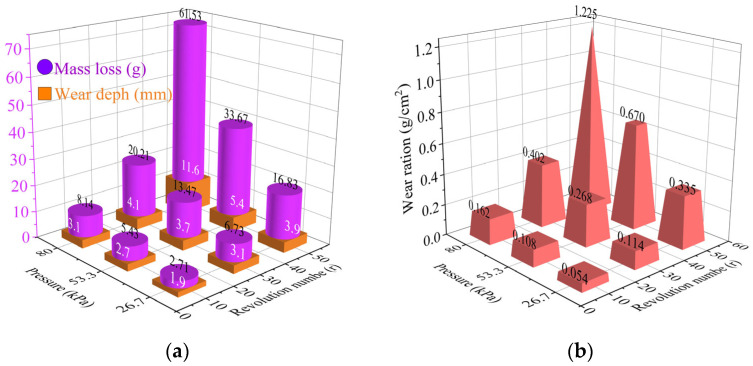
Wear erosion result of EICP treated specimens. (**a**) mass loss and wear depth; (**b**) wear rate.

Figure 12 demonstrates that the soil crust remains intact under specific conditions: at 80 kPa pressure with revolution numbers below 30 circles, or at 53.3 kPa pressure with revolution numbers below 50 circles. The maximum values for mass loss and wear depth were 33.67 g and 5.4 mm, respectively, under each condition. The maximum wear rate recorded was 0.670 g/cm^2^. Exceeding these critical friction conditions compromises the solidification layer. Here, the insufficient strength of the unconsolidated silt beneath the soil crust fails to resist the abrasion from the rubber friction head, resulting in a rapid increase in wear depth and mass loss. The experimental findings indicate that the soil solidification layer withstands repeated abrasion from fully loaded trucks for 30 cycles and unloaded trucks for 50 cycles.

## 3. Conclusions

This investigation into the potential of enzyme-induced carbonate precipitation (EICP) to reduce wind erosion of Yellow River silt revealed significant results. The bio-gel formation through EICP was found to enhance the bearing capacity, wind–rain erosion resistance, and wear resistance of the silt through the in situ generation of a mineral gel matrix. The level of improvement was notably affected by the concentration of the cementation solution and the spraying volume.

(1) The optimal cementation solution, consisting of equal concentrations of urea and CaCl_2_ (1.25 mol/L) applied at 4 L/m^2^, enabled controlled gelation through sequential spraying (first urea-CaCl_2_ solution, then urease).
(2) This protocol produced a 14.9 mm thick bio-cemented gel layer with compressive strength exceeding 752 kPa, where CaCO_3_ crystallization created a self-reinforcing gel architecture.
(3) The gel-stabilized crust maintained integrity under Beaufort scale 7 winds (51–61 km/h), resisted 40 mm/h rainfall erosion, and endured 30 cycles of fully loaded truck abrasion.

These findings establish EICP as a novel bio-gel technology that strengthens silt via natural gel-phase cementation, providing an effective biological solution for dust mitigation in the Yellow River region.

Limitations and Future Work:

(1) Our tests focused on short-term performance; long-term durability under freeze–thaw cycles and microbial degradation needs validation.
(2) Field-scale cost analysis (USD 0.32/m^2^ for lab trials) should be conducted to assess economic viability.
(3) Combining EICP with biopolymers may further improve erosion resistance in high-shear environments.

## 4. Materials and Methods

### 4.1. Materials

#### 4.1.1. Yellow River Silt

**Figure 13:**
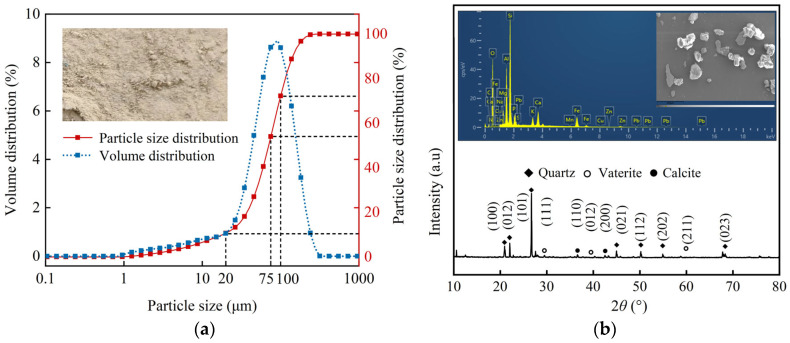
Particle size and microscale identification of the test silt: (**a**) particle size distribution and grain gradation; (**b**) microscopic view of the silt with the XRD and SEM images.

The silt used in this experiment was collected from Zhengzhou City in the central Yellow River alluvial plains, Province of Henan, China. The D_10_, D_50_, and D_90_ values of the silt are 75 μm, 91 μm, and 158 μm, respectively. Figure 13a presents a depiction of the distribution of particle sizes in the soil that was utilized. X-ray diffraction analysis identified quartz as the predominant mineral and SiO_2_ as the compound within the silt, as shown in Figure 13b. Detailed physical properties of the soil can be found in Table 2.

#### 4.1.2. EICP Solution

**Figure 14:**
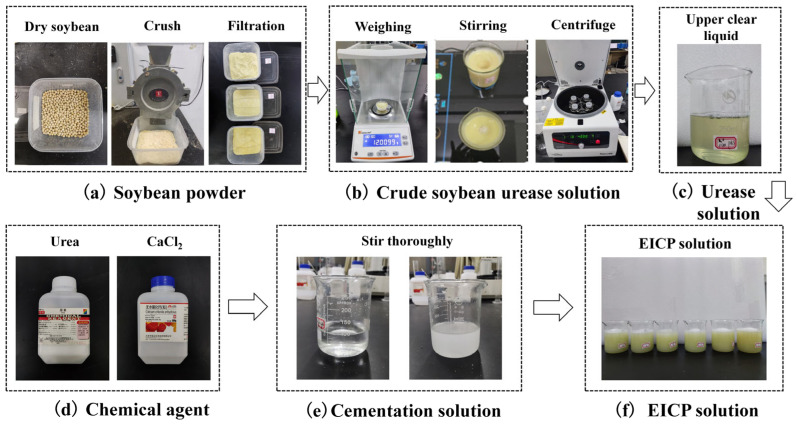
Schematic steps for preparing EICP solution: (**a**) soybean powder; (**b**) initial solution; (**c**) urease solution; (**d**) chemical agent; (**e**) cementation solution; (**f**) EICP solution.

The EICP solution consists of soybean urease solution (US) and cementation solution (CS). The bean powder was sifted into various particle sizes: 1.5, 1.0, 0.75, 0.5, 0.25, and 0.075. Subsequently, the powder was mixed with PBS liquid at mass ratios of 1:40, 1:30, 1:20, 1:10, and 1:5. After thorough stirring, the mixture was centrifuged at 3000 rpm for 15 min, and the clear liquid at the top was collected to obtain the soybean urease solution. These solutions were then adjusted to pH levels ranging from 1 to 13 and stored at temperatures of 4 °C and 25 °C for urease activity analysis. The selection of 4 °C and 25 °C reflects standard enzymatic storage (4 °C) and application (25 °C) conditions, where 25 °C represents a practical ambient baseline for activity comparison, while 4 °C minimizes enzymatic degradation without inducing freezing damage, as validated in prior soybean urease studies [9,10,11]. The cementation solution primarily consists of urea and calcium chloride, with concentrations maintained between 0.25 mol/L and 1.5 mol/L. The soybean urease solution and cementation solution are combined in ratios of 1:2, 1:1.5, and 1:1 to create the EICP solution, as depicted in Figure 14.

#### 4.1.3. Specimens

This EICP solution comprises urease, urea, and calcium chloride solutions, which were sprayed onto the silt surface in varying volumes and sequences. Subsequently, the disk-shaped specimens, with a diameter of 200 mm and a height of 30 mm, and square disk-shaped specimens, with a side length of 300 mm and a height of 40 mm, were placed in a constant temperature box with curing at 25 °C. In order to meet the requirements of rainwater infiltration, square specimens should be prepared by drilling holes in the bottom of the mold and laying geotextiles.

### 4.2. Test Methods

#### 4.2.1. Urease Activity Measurement

Since the changes in the amount of conductivity of the urease solution are directly proportional to urea hydrolysis, the urease activity can be determined using the amount of hydrolyzed urea conductivity method. Specifically, 3 mL of urease solution was mixed with 27 mL of urea solution at a concentration of 1.11 mol/L. The changes in conductivity of the solution were then monitored for the initial 30 min at 25 °C. The urease activity can be obtained based on the conductivity change using the following equation proposed by Whiffin [33]:

(3) $$U=k\frac{\Delta \sigma }{\Delta t}F$$

where U is the urease activity (mmol/min); Δσ is the difference in conductivity (ms·cm^−1^·min^−1^); Δt is the time (min); F is the dilution factor of urease solution, taken as 10 in this experiment; k is the relationship between solution conductivity and moles of ammonium ions, taken as 11.11 based on reference [34].

#### 4.2.2. CaCO_3_ Content and Conversion Rate Measurement

In this experiment, the urease activity was measured using the conductivity meter method, and the CaCO_3_ content of the samples was determined via EDTA titration method. First, the treated soil specimen was rinsed with deionized water to remove the residual soluble substances. After that, the specimen was dried and placed in an excess amount of hydrochloric acid (HCl) solution with a concentration of 1 mol/L to dissolve all the generated CaCO_3_ precipitates. The Ca^2+^ content dissolved in the hydrochloric acid solution was measured using titration method. Then, the CaCO_3_ content in the specimen can be determined based on the measured Ca^2+^ content. CaCO_3_ conversion rate was defined as the ratio of actual CaCO_3_ production to theoretical CaCO_3_ production.

#### 4.2.3. Spraying Scheme

The recommended spraying volume of dust suppressor in sandy soil ranges from 3.66 to 4.00 L/m^2^ [11]. Since silt particles are smaller than sand particles, it is necessary to determine the appropriate spraying volume for silt sand in Zhengzhou. For this experiment, the designed spray volume of EICP solution for this experiment was as follows: 1 L/m^2^, 2 L/m^2^, 3 L/m^2^, 4 L/m^2^, and 5 L/m^2^.

The curing effect of EICP dust suppressor solution for silt soils will be influenced by the order of mixture and spraying. In the test, the order of spraying was, therefore, divided into three types, namely working conditions W1, W2, and W3, as shown in Table 2.

① Urease solution and urea solution first, followed by calcium chloride solution (W1);
② Calcium chloride solution and urea solution first, followed by urease solution (W2);
③ Direct mixing and spraying (W3).

#### 4.2.4. Performance Tests

Performance tests include the unconfined compressive strength test, wind–rain resistance test, and friction test of plate samples.

1. Wind–rain resistance test

The wind force is typically classified into 13 levels, ranging from 0 to 12. Based on the wind data for Zhengzhou between 2011 and 2023, it can be observed that the prevailing wind conditions were predominantly at level 3, which corresponds to a wind speed of approximately 5.4 m/s. In order to simulate realistic wind erosion in this experiment, this experiment set the wind speeds at three different levels: 5 m/s (level 3), 10 m/s (level 5), and 15 m/s (level 7). Each experiment will last for a duration of 60 min.

**Figure 15:**
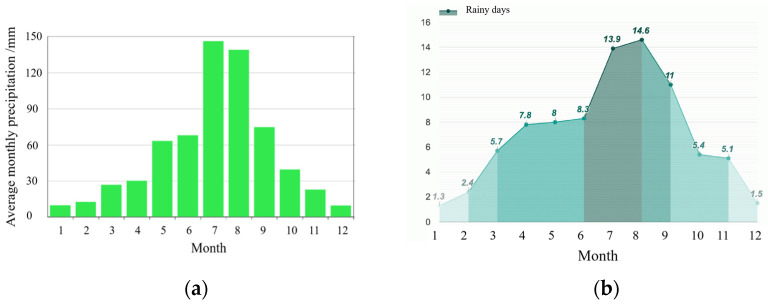
Precipitation date from Zhengzhou: (**a**) average monthly precipitation; (**b**) average rainy days.

According to data from the Central Meteorological Bureau, Figure 15 illustrates the monthly average rainfall and number of rainy days in Zhengzhou. The annual average precipitation in the city ranges from 477.08 to 1167.30 mm, with monthly averages of rainy days varying between 1.3 and 14.6 days. July and August have the highest precipitation levels, with recorded amounts of 146.2 mm and 138.9 mm, respectively. Therefore, a rainfall level of 40 mm is considered relatively high in the Zhengzhou area. This experiment simulated three different intensities of rainfall: 7 mm/h, 15 mm/h, and a heavy downpour at a rate of 40 mm/h for a duration of one hour.

This experiment involves a cyclic erosion process with wind–rain–wind action lasting 1 h each. The wind speeds were 5, 10, and 15 m/s (equivalent to Beaufort scale 3, 5, 7), while the precipitation rates were set at 7, 15, and 40 mm/h (representing heavy rain, rainstorm, and heavy rainstorm). Working conditions WR1 to WR3 represent increasing values of wind speed and precipitation rates from low to high. The untreated silt and specimens sprayed with deionized water were set as the control group, and the design plan is provided in Table 3.

**Figure 16:**
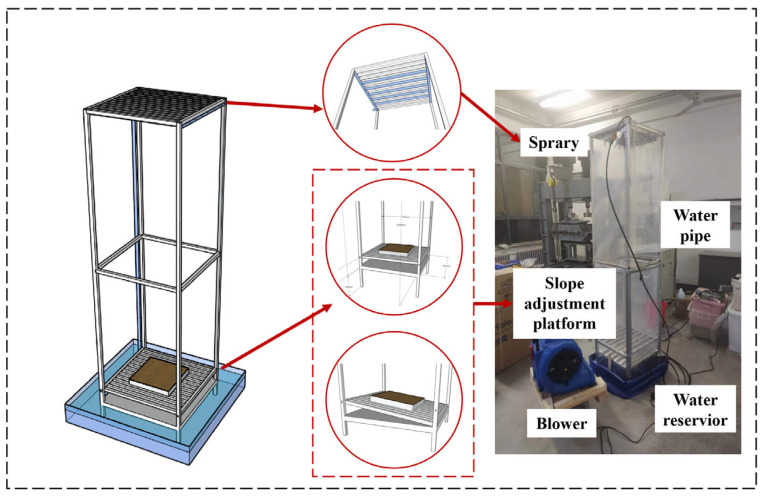
Self-designed wind–rain erosion test.

A self-designed wind–rain erosion test device was utilized (Figure 16), comprising a blower, a water reservoir, a sample disk, an atomizing nozzle, a slope-adjusting platform, and a water-collecting tank. The atomizing nozzle is connected via the catheter, diaphragm pump, and flow meter to simulate various levels of rainfall intensity during testing. The water storage tank is positioned beneath the bottom plate for rainwater recycling purposes. Externally to the device, the blower and anemometer are employed to regulate different wind speeds.

2. Friction test

In evaluating the abrasion resistance of the solidification layer by EICP, this study utilized the UMT-2 micro friction tester to simulate the friction caused by truck rubber tires on the ground. The UMT-2 (Universal Mechanical Tester-2, Bruker Corporation, Billerica, MA, USA), a tribological testing system, was employed to simulate rubber tire-ground friction under controlled loads and rotational speeds. Its reciprocating ball-on-disk configuration mimics real-world abrasion mechanisms, aligning with ASTM G133-22 standards [1]. The UMT-2′s precision (±0.1% load accuracy, ±1 rpm speed control) ensures reliable quantification of the solidification layer’s wear resistance.

According to the site conditions, the maximum pressure exerted by fully loaded trucks on the road surface through tires is 80 kPa, equivalent to applying a load of 16.07 N on the lever arm of the micro friction tester. Meanwhile, the maximum driving speed of vehicles is 5 km/h when entering and leaving the factory or the production site, corresponding to a rotational speed of 2.19 r/s on the micro friction wear tester. Therefore, the rotate speed was set to 120 rpm, and the loads were 5 N, 10 N, and 15 N, with 10, 30, and 50 cycles for the wear tests, respectively. The details of the wear test design can be found in Table 4.

### 4.3. Microscopic Evaluation

In this study, the Hitachi Regulus 8230 field-emission scanning electron microscope (Hitachi Corporation, Japan, Tokyo) was used to observe the morphological characteristics of mineral precipitates in the silt after EICP treatment at different magnifications. The samples were ground into a fine powder using a mortar and pestle, and the crystalline phases of the powder were identified using the Malvern Panalytical Empyrean X-ray diffractometer (Global Headquarters, Malvern Panalytical, Malvern, UK), with a diffraction angle range from 10° to 80° and a scan rate of at least 10°/min.

## Figures and Tables

**Table 1:** The design of mixing sequences.

Working Conditions	Spray Bottle I			Spray Bottle II		
	Urea Volume/mL	Calcium Chloride Volume/mL	Urease Volume/mL	Urea Volume/mL	Calcium Chloride Volume/mL	Urease Volume/mL
W1	30	0	60	0	30	0
W2	30	30	0	0	0	60
W3	30	30	60	0	0	0

**Table 2:** Basic properties of test silt.

Density ρ/g·cm^−3^	Water Content W_op_/%	Void Ratio e	Maximum Dry Density ρ/g·cm^−3^	Specific Gravitys G_s_/g·cm^−3^	Permeability Coefficient K_T_/cm·s^−1^
1.39	10.34	1.14	1.26	2.7	2.5 × 10^−4^

**Table 3:** The design of wind–rain erosion test.

Working Conditions	Rotation 1		Rotation 2		Rotation 3	
	Wind Speed *V*_1_/m·s^−1^	Time *t*_1_/min	Rainfall *R*_2_/mm·h^−1^	Time *t*_2_/min	Wind Speed *V*_3_/m·s^−1^	Time *t*_3_/min
WR1	5	60	7	60	15	60
WR2	10	60	15	60	15	60
WR3	15	60	40	60	15	60

**Table 4:** The design of wear test.

Working Conditions	Load (N)	Pressure (kPa)	Revolution (r)
L5–r10	5	26.7	10
L5–r30	5	26.7	30
L5–r50	5	26.7	50
L10–r10	10	53.3	10
L10–r30	10	53.3	30
L10–r50	10	53.3	50
L15–r10	15	80	10
L15–r30	15	80	30
L15–r50	15	80	50

## Data Availability

The data used to support the findings of this study are available from the corresponding author upon request.
